# An Environmental Scan of Services for Adolescents and Young Adults Diagnosed with Cancer Across Canadian Pediatric and Adult Tertiary Care Centres

**DOI:** 10.3390/curroncol33020068

**Published:** 2026-01-24

**Authors:** Nicole Rutkowski, Sara Beattie, Fiona Schulte, Chantale Thurston, April Boychuk, Marie de Guzman Wilding, Chana Korenblum, Perri R. Tutelman

**Affiliations:** 1 School of Psychology, Faculty of Social Sciences, University of Ottawa, Ottawa, ON K1N 6N5, Canada; 2 Psychosocial Oncology, Arthur J.E. Child Comprehensive Cancer Centre, Calgary, AB T2N 5G2, Canada; 3 Department of Oncology, University of Calgary, Calgary, AB T2N 4N1, Canada; 4 AYA CAN—Canadian Cancer Advocacy, Winnipeg, MB R3X 2E3, Canada; 5 Division of Adolescent Medicine, Hospital for Sick Children Toronto, Toronto, ON M5G 1X8, Canada; 6 Department of Supportive Care, Princess Margaret Cancer Centre, Toronto, ON M5G 2C4, Canada; 7 Department of Psychology, University of Calgary, Calgary, AB T2N 1N4, Canada

**Keywords:** adolescents and young adults, cancer, clinical care, supportive care

## Abstract

Adolescents and Young adults (AYAs: 15–39 years) diagnosed with cancer face unique medical and psychosocial challenges requiring specialized care. This study conducted an environmental scan of AYA-specific programming and services currently offered across Canadian tertiary care centres. Key informants from pediatric and adult cancer centres in Canada reported on program logistics, AYA specialized staff and training opportunities, and collaboration between centres, funding, and specific areas of interest for AYA care such as palliative care, fertility, fatigue, and sexual health. Surveys were completed by 13/16 (81%) pediatric sites and 19/23 (83%) adult sites. Only about half of pediatric sites (*n* = 8/13) and adult sites (*n* = 9/19) who responded reported offering any AYA-specific cancer services or programming. One third of centres without programming reported to be working on developing programming. Only 6 sites reported to offer specialized AYA training. Several barriers were reported, such as the need for collaboration among institutions and improvement of oncofertility services. Significant disparities exist regarding geographic availability of services, the range of services available, and the populations served. Findings will guide researchers, health professionals, and provincial health authorities in the development of highquality and equitable services and programs for AYAs diagnosed with cancer across Canada.

## Simple Summary

Only about half of Canada’s major pediatric and adult cancer hospitals offer specialized programsfor young people with cancer. Available services include support for families and caregivers, guidance on returning to work or school, fertility and sexual health support, and palliative care. Some hospitals are developing programs, such as informational websites for young patients, education for healthcare providers, sexual health resources, and hiring staff trained in adolescent and young adult (AYA) care, though training and support remain limited. Collaboration between pediatric and adult hospitals occurs in some areas; however, it is often hindered by costs, limited training, challenges across multiple sites, and lack of management support. Most programsrely on private donations. The study highlights wide regional variation in service availability, meaning young people may have many, few, or no specialized support depending on where they live in Canada.

## 1. Introduction

In 2023, an estimated 9000 adolescents and young adults (AYAs; 15–39 years) were diagnosed with cancer in Canada and this number is predicted to increase over the coming years [1,2]. AYAs are navigating a developmentally complex period of life and face unique psychosocial challenges when diagnosed with cancer [3], such as interruptions to work and school, romantic relationships/sexuality, parenting, peer relationships, fertility, and finances [4,5]. Medically, the underlying biology of malignant disease in AYAs appears to be distinct from other age groups for many tumour types and remains poorly understood [4,6]. In Canada, the healthcare system is publicly funded but provincially administered, which has led to interprovincial variations in the organization and delivery of care. AYAs are treated at either pediatric or adult sites depending on age (<18 years are generally treated at pediatric sites and >18 years are generally treated at adult sites), creating significant barriers for offering specialized training for staff, appropriate programming, and access to clinical trials [7,8]. AYAs face poorer medical and psychosocial outcomes compared to pediatric and adult populations [4,9]. Improvements in health and psychosocial outcomes have been reported when AYAs receive developmentally tailored programming and care [10,11,12,13]. However, specialized care for AYAs is not universally available across Canada [14]. These inequalities are not unique to Canada, with other countries reporting similar challenges [15,16,17,18].

In the last two decades, there has been an increased recognition of AYAs’ unique needs, including in Canada, where efforts have been made to improve AYA care [19]. From 2008 to 2016, the Canadian Task force on Adolescents and Young Adults with Cancer offered recommendations including provision of age-appropriate psychosocial, survivorship, palliative, and medical care services, as well as increasing access to clinical trials and reducing inequities in care [20]. Since the release of the report, numerous important initiatives have been undertaken across the country including the Canadian framework for the care of AYAs [21], the establishment of provincial clinical practice guidelines [22], AYA system performance reports [23], AYA oncology becoming a recognized area of specialization by the Royal College of Physicians [24], increasing access to oncofertility services [25,26], patient-led research priority setting [27], development of community programs [28], and patient advocacy groups [29]. These efforts have led to the establishment of dedicated AYA programs across some cancer centres in Canada [30,31,32].

However, despite two decades of advancements and significant momentum, AYAs in Canada continue to report unmet needs and experience significant geographical disparities in access to services [14,33]. In 2011, Ramphal and colleagues conducted an environmental scan of AYA programming across Canadian hospitals and found a lack of AYA-specific services, resources, collaborative activities, and staff across both pediatric and adult cancer centres [14]. At the time, 44% of pediatric sites reported offering AYA specific programs to address school-related concerns, 42% of adult sites reported offering AYA-specific health education materials, and 25% of sites reported offering AYA only support groups at both adult and pediatric sites. Given significant initiatives to improve AYA programming over the last decade, the goal of this study was to examine the current landscape of AYA programming and services that are being offered in Canadian pediatric and adult cancer centres and whether gaps continue to exist.

## 2. Materials and Methods

This study assessed the current Canadian landscape using a survey-based environmental scan. Stemming from naturalistic studies, this methodology scans the environment for existing information and resources and can include interview, surveys, observation, and interpretation of documents [34]. Environmental scans can be utilized to facilitate more informed decision-making, support the allocation of appropriate resources, and shed light on disparities in healthcare services [34]. In this study, an environmental scan was used to assess the state of AYA programming across Canada and report advancements in AYA programs and services over the last decade, as well as continued persisting gaps in services. Pediatric and adult hospitals that provide comprehensive cancer care, research, and support for patients with cancer were invited to participate. This environmental scan was conducted as part of a program evaluation to assess existing programming being offered throughout Canada with the goal to improve clinical services delivery for AYAs within Alberta. Formal ethics approval was deemed not necessary given this study was a quality improvement project, did not involve direct contact with patients, and posed minimal risk. Consent for participation was obtained from key informants at the start of the survey.

### 2.1. Cancer Centre Identification

Sixteen pediatric cancer centres were identified through the C17, an organization composed of the institutionally appointed heads of the sixteen pediatric hematology, oncology, and stem cell transplant programs across Canada. For adult cancer centres, twenty-three tertiary and quaternary cancer centres were identified through internet searches of provincial oncology programs (see Table 1 for the full list of centres contacted). Regional and community hospitals were not approached for this study, apart from a regional hospital in the Yukon, as the province does not have a tertiary site and would otherwise not be represented in the data. A key informant with clinical and/or administrative knowledge of AYA cancer services was identified for each cancer centre to increase the probability of accurate and complete responses. Key informants at each centre were identified through existing networks and contacting hospital administration.

### 2.2. Data Collection

The research team developed a survey to assess AYA services in pediatric and adult cancer centres. The survey built upon the domains assessed by Ramphal et al. (2011) [14]. The survey was translated from English into French to facilitate data collection in both languages. The questionnaire included items assessing program logistics (i.e., cities served, language of services offered, designated AYA spaces), AYA-specialized staff and training opportunities, collaboration between pediatric and adult centres, distress screening, funding sources, and specific AYA resources or services for palliative care, fertility, caregiver support, sexual health, and clinical trials. The questionnaire was piloted by a manager and AYA nurse navigator at a local cancer centre in Calgary, Alberta to ensure usability. The questionnaire was found to be acceptable, and no significant changes were made to the questionnaire. A copy of the questionnaire is available in the Supplementary Materials.

A personalized, tailored email with the online survey link was emailed to an identified key informant requesting completion of the survey on behalf of their institution or identification of another individual who may be best positioned to complete the survey. Questionnaires were sent to contacts at 23 adult tertiary hospitals and 16 pediatric hospitals that provide cancer care. Survey responses were collected between June 2024 and April 2025. If no response was recorded after 2–4 weeks, reminder emails were sent. The research team tracked which hospitals responded and attempted to identify an alternate key informant if no response was received after four weeks.

### 2.3. Data Analysis

A descriptive analysis was conducted to synthesize information on the AYA programs available and the characteristics of services available. Qualitative findings were limited and were summarized narratively to add additional information on services available and perceived barriers.

## 3. Results

### 3.1. Respondent Demographics

**Figure 1:**
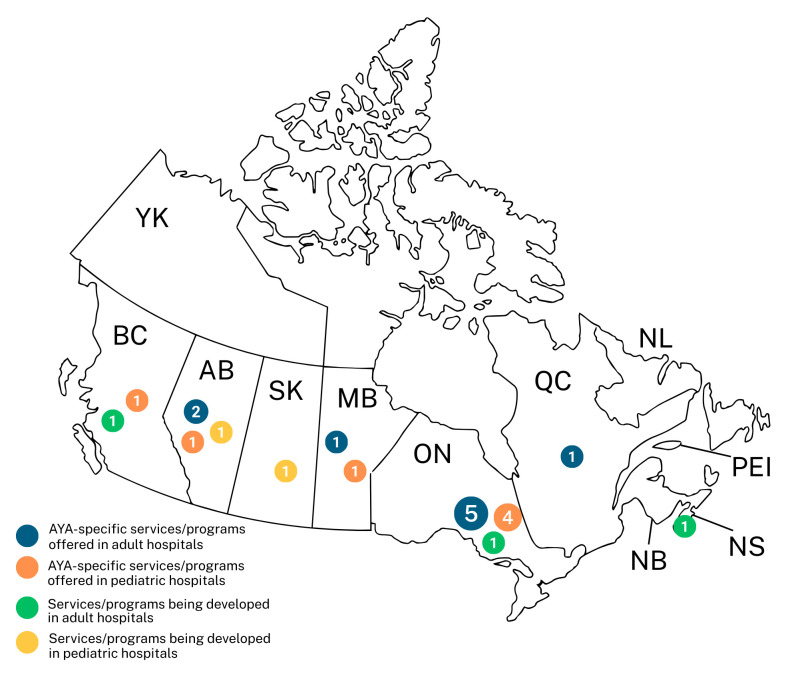
AYA-specific services and programs across Canada.

A total of 32 completed surveys were received from 13/16 (81%) pediatric sites and 19/23 (83%) adult hospitals from across Canada (see Figure 1). Surveys were completed by a variety of healthcare and management professionals. For pediatric settings, the survey was completed by oncologists (*n* = 4; 30%), psychologists (*n* = 3; 23%), nurses (*n* = 2; 15%), managers (*n* = 2; 15%), social workers (*n* = 1; 8%), and nurse practitioners (*n* = 1; 8%). For adult cancer centres, the survey was completed primarily by nurses (*n* = 6; 32%), managers (*n* = 5; 26%), oncologists (*n* = 2; 11%), a physician assistant (*n* = 1; 5%), psychologist (*n* = 1; 5%), nurse practitioner (*n* = 1; 5%), researcher (*n* = 1; 5%), palliative care physician (*n* = 1; 5%), and social worker (*n* = 1; 5%); see Table 2 for the full demographic list. Significant attempts were made to reach key informants at Quebec hospitals with limited success, including emailing several identified individuals in both French and English, calling hospitals directly, inquiring through research institutions affiliated with teaching hospitals, and leveraging existing contacts.

### 3.2. Available AYA Services and Programs

Among the 32 centres that responded to the survey, 54% pediatric hospitals (*n* = 7/13) and 47% adult hospitals (*n* = 9/19) reported offering some degree of AYA-specific services and programming. AYA-specific services and programming was available in Alberta, British Columbia, Manitoba, Ontario, and Quebec. No AYA-specific programming was available in New Brunswick, Newfoundland, Nova Scotia, Prince Edward Island, Saskatchewan, and Yukon. Among centres that indicated they did not have any AYA-specific programming, key informants were asked whether AYA-specific resources and programs were being developed at their site. Three out of ten (30%) adult centres and two out of six (33%) pediatric centres reported working on developing AYA-specific programming (Figure 1). Programs and resources being developed included websites with AYA resources, oncofertility referral pathways, patient and healthcare provider AYA resources, sexual health and supportive care services for teens with cancer, and hiring additional AYA-specific staff. Reasons for not developing programs included limited AYA patients served, lack of resources and time, needing to develop partnerships, other issues being prioritized, and COVID-19 impacts on program delivery.

Additionally, respondents were asked about the priority of AYA programming and service development; 11 (92%) adult centres and 7 (88%) pediatric centres indicated AYA program and service development was a priority at their institution. Respondents indicated that AYA services were prioritized at their institutions through steering committees, advocacy from dedicated health care providers and medical leads, increased funding/resources for AYA care improvement including dedicated provincial funding, recognition of AYA needs among leadership, management, and provincial governments, and AYA-specific programming and staff (i.e., multidisciplinary teams).

### 3.3. Institutional Age Limits for AYA Programming

From the sites that reported offering or developing AYA-specific programs, adult centres (*n* = 11) reported the lowest age limit was 15 years (*n* = 5), 17 years (*n* = 2), 18 years (*n* = 4), and 19 years (*n* = 1), and the upper age limit to access services was 39 years (*n* = 10) and 40 years (*n* = 1). Among pediatric centres (*n* = 9), the lowest age limit reported was 13 years (*n* = 5), 14 years (*n* = 1), and 15 years (*n* = 3), and the upper age limit was 18 years (*n* = 5) or greater than 18 years (19–35 years; *n* = 4).

### 3.4. Available Programs and Services for AYAs

Regarding AYA-specific programming and services, centres most commonly reported offering AYA support groups (*n* = 8), AYA patient navigation (*n* = 7), information packages about community resources (*n* = 7), and individual counselling for AYAs (*n* = 10), with a few centres reporting educational classes/webinars (*n* = 4), a newsletter (*n* = 3), social media account (*n* = 2), and a newly diagnosed class (*n* = 1). The majority of centres who reported offering AYA services also reported offering services for caregivers and family of AYAs (*n* = 17; 10 adult, 7 pediatric). Available services for family and caregivers included AYA navigation, individual counselling support for parents (but not siblings), access to social work and/or psychology counselling services, support groups, a caregiver clinic, and specific tumour group services for caregivers of patients with leukemia or breast cancer.

**Screening for distress.** Eleven centres (6 adult; 5 pediatric) reported routinely screening AYAs for distress. The most common screening method reported was the Edmonton Symptom Assessment System (*n* = 6). Other screening tools included the Distress Thermometer, Canadian Problem Checklist, and screening performed by social work or nurses.

**Return to work/school**. For return to work and school, 12 centres (6 adult; 6 pediatric) reported to offer specific services or programs for AYAs. Services included navigators, counselling with a social worker, interlink nurse, school–teacher liaison, or work/school transition counsellor, access to occupational therapy, and adolescent-specific return to school planning.

**Fertility**. For specific fertility services and programming, 16 centres (9 adult; 7 pediatric) indicated they offered services to address fertility concerns and preservation options for AYA patients. Thirteen centres (8 adult; 5 pediatric) reported that fertility preservation options were discussed with all AYA patients, most often by an oncologist or nurse. For pediatric centres, five sites reported discussing fertility preservation options with AYAs under the age of 18. Types of services offered included resources on oncofertility (*n* = 16) and referral pathways to fertility clinics (*n* = 15), followed by individual counselling with psychology (*n* = 8), social work (*n* = 7), or a clinical nurse specialist (*n* = 4). Other services reported included an educational class on oncofertility (*n* = 2) and collaboration with a reproductive centre affiliated with the hospital (*n* = 1). No centres reported offering support groups around fertility. Barriers to offering fertility services reported were time, costs for patients, lack of knowledge, discomfort among healthcare providers and perceived discomfort among patients/families, limited access to fertility preservation for women, no specific programming available, inadequate referral pathways, inconsistent practices among healthcare providers, limited access to fertility clinics in the region, and urgency to start treatment. Current existing gaps reported qualitatively by centres included no local fertility clinics available, access to timely resources, no standard referral process or process to identify AYA patients, lack of funding, and lack of training offered around fertility and preservation options for healthcare providers, specifically for nurses who may frequently counsel AYA patients on fertility concerns.

**Sexual health**. Eleven sites (7 adult; 4 pediatric) reported to have specific sexual health services or programs for AYAs. Types of services and programs included resources on sexual health (*n* = 5), individual counselling with a clinical nurse specialist (*n* = 5), an educational class (*n* = 3), individual counselling with social work (*n* = 3), psychology (*n* = 2), a sexual health consultant (*n* = 2), physician assistant (*n* = 1), or gynecologist (*n* = 1).

**Palliative care**. Ten sites (4 adult; 6 pediatric) reported specific services or programs for palliative care, which included resources on end of life (*n* = 10), support groups (*n* = 3), individual counselling social work (*n* = 3), psychology (*n* = 3), spiritual counselling (*n* = 6), an AYA palliative care clinic (*n* = 2), AYA-specific palliative care physicians, Pediatric Advanced Care Team support, and individual support with a clinical nurse specialist. Some resources referenced included Voicing My Choices, Living out Loud, and Children’s Hospice Care.

**Fatigue**. Six sites (4 adult; 2 pediatric) reported specific services to address cancer-related fatigue and 9 sites (8 adult; 1 pediatric) reported routinely screening for cancer-related fatigue. Specific services included resources on cancer-related fatigue (*n* = 4), individual counselling with social work (*n* = 3), an educational class (*n* = 2), vocational/return to work counselling (*n* = 2), individual counselling with a psychologist (*n* = 1), a rehabilitation clinic, and occupational therapy. Resources included e-learning modules, online videos, and education materials, however, one site noted these were not AYA-specific.

### 3.5. Collaboration Between Pediatric and Adult Centres and Specialized Staff

Regarding the collaboration between pediatric and adult centres, 14 centres (9 adult; 5 pediatric) reported offering services for AYAs in collaboration with another centre and 9 centres (6 adult; 3 pediatric) reported having cross-appointed staff across settings. Collaborative activities included offering programming (i.e., support groups), sharing educational resources, offering support during transitioning services, and research/clinical trial enrollment. Two centres reported actively working on creating collaborations between pediatric and adult sites. Cross-appointed staff included nurses, radiation oncologists, oncologists, and psychosocial oncology providers.

Barriers to collaboration were primarily funding (*n* = 16), training (*n* = 12), logistics of having staff work between different institutions (*n* = 12), and lack of organizational support (*n* = 9). Other barriers reported included environmental (i.e., rural setting) (*n* = 2), limited AYA patients, lack of knowledge of collaboration or services offered at other sites, and difficulty around consent with younger patients. One site reported experiencing no barriers to collaboration. Only 5 sites (3 adult; 2 pediatric) reported having specialized training on caring for AYA patients, while 15 centres reported not having any training available. The specialized training described was primarily for nurses. The training mentioned included education through the Cactus Cancer Society and DeSousa Institute.

For AYA-specific staff, 11 centres (9 adult; 2 pediatric) reported employing AYA-specific staff at their sites. AYA-specific staff included a clinical nurse specialist (*n* = 5), medical director (*n* = 3), social worker (*n* = 3), physician (*n* = 3), research staff (*n* = 3), AYA patient navigator (*n* = 3), program coordinator (*n* = 2), clinical psychologist (*n* = 2), school/work transitions counsellor (*n* = 2), psychiatrist (*n* = 2), neuropsychologist (*n* = 1), as well as some sites reporting a physician assistant, occupational therapist, fertility specialist, and dietitian.

### 3.6. Communication with AYA Patients

The most common communication tool used by centres to communicate about programming and services with AYA patients was email (*n* = 14), followed by phone (*n* = 10), website (*n* = 10), newsletter (*n* = 5), and social media (*n* = 2). Other methods included automatic referral pathways, communication from healthcare providers during in-person appointments, posters, hardcopy teaching packages, or no established means of communication. Available programs were primarily offered in English (*n* = 13), or mostly English with some services offered in French (*n* = 5). Only two sites, one located in Ontario and one in Quebec, reported offering services in both languages equally.

### 3.7. Funding for AYA Services and Programs

AYA-specific programming and services were reported to be primarily funded through philanthropic donations (*n* = 9, 7 adult; 2 pediatric), provincial healthcare funds for the provision of AYA services (*n* = 5, 3 adult; 2 pediatric), and research grants or government grants (*n* = 4, 2 adult; 2 pediatric). Some centres also reported being funded primarily through healthcare funds and supplemented by donations (*n* = 2, pediatric) or primarily funded by donations and supplemented by healthcare funds (*n* = 2, pediatric) or did not have any funding (*n* = 1, adult). Several centres reported not knowing where funding originated (*n* = 5, 1 adult; 4 pediatric).

### 3.8. AYA-Specific Space

A few centres reported offering AYA-specific space; 5 centres (1 adult, 4 pediatric) reported having an inpatient AYA space and 3 sites (1 adult; 2 pediatric) reported having an outpatient AYA space. Inpatient spaces were described as a room for family/support, a teen room or lounge, and an AYA lounge or space. Outpatient spaces included an AYA group room and an AYA/teen lounge. However, it was noted by one site that the outpatient teen room was not teen-friendly and frequently used by other populations.

### 3.9. Accessing Clinical Trials for AYAs

Barriers for clinical trials included difficulty identifying trials for rare cancers (*n* = 8, 6 adult; 2 pediatric), clinical trials not available (*n* = 6, 2 adult; 4 pediatric), lack of time (*n* = 4, 2 adult; 2 pediatric), or lack of knowledge around clinical trials (*n* = 2, 1 adult; 1 pediatric). One centre reported no barriers around accessing clinical trials due to affiliation with a large research centre. However, six centres reported being unsure of what barriers may exist at their institutions.

## 4. Discussion

In this environmental scan of AYA programs and services in Canada, approximately half of the sites surveyed reported offering AYA-specific services and programs and a third reported working on developing AYA programming or services. Despite significant initiatives to improve AYA programming and services, service provision and availability of programming vary considerably across Canada. Geographic disparities continue to exist, with the majority of AYA programming being available in Ontario, Alberta, and Manitoba. Some degree of programming is available in British Columbia and Quebec; however, no programs or services are currently available in New Brunswick, Newfoundland, Nova Scotia, Prince Edward Island, Saskatchewan, or the Yukon. Among these, only Saskatchewan and Nova Scotia reported to be working on developing any sort of AYA-specific programming or services. These findings highlight a concerning disparity for Canadian AYAs diagnosed with cancer.

In the 15 years since the last review of AYA programs in Canada, improvements appear to have been achieved within palliative care in pediatric settings for AYAs, oncofertility and sexual health services in both pediatric and adult hospitals, and return to work/school services in adult hospitals (see Table 3) [14]. One of the recommendations from the past study was to prioritize collaboration between adult and pediatric sites [14]. However, collaboration between institutions appears to continue to be a challenge. This study found that 32% of adult cancer centres and 23% of pediatric centres reported cross-appointed staff, which is a similar finding to the last environmental scan [14]. Recent studies demonstrate that cross-appointed staff and collaboration between centres can provide better health outcomes to meet the unique needs of AYAs and play a critical role in AYA program success [35]. Increasing efforts in collaboration between pediatric and adult hospitals continues to be an important priority and future studies may wish to focus on the identification of barriers and enablers to this collaboration at Canadian hospitals.

One outcome that would benefit substantially from improved collaboration across pediatric and adult sites is access to clinical trials. Indeed, advancements in cancer therapies to improve survival among AYAs have lagged behind pediatric and older adult populations, with poorer participation in clinical trials likely being one contributing factor to the divide [36]. Several barriers were reported for enrollment in clinical trials, including identifying trials for rare cancers, clinical trials not being available at the site, lack of time, or lack of knowledge around clinical trials, which align with the literature [35,36]. These are important, ongoing barriers to further examine and address to increase access to clinical trials for AYAs in Canada.

While few countries have national AYA cancer care policies, government support has played a key role in program success, such as the CanTeen program based in Australia [37]. In this study, key informants reported increased provincial funding for AYA-specific programming and services; however, the majority reported funding programs and services through philanthropic donations. While it is encouraging to see growing support for AYA services, reliance on philanthropic funds is not sustainable. To achieve more equitable and consistent services across Canada, allocated provincial funding for the provision and development of AYA services is needed, as well as funding for the development of specialized AYA training programs. Presently, only 16% of adult sites and 15% of pediatric sites reported to offer specialized AYA training, suggesting limited training opportunities available to healthcare providers across Canada.

Another important consideration may be the inclusion of AYAs in the co-design of more sustainable programming that meets the needs of end users. Although the present study did not assess whether AYAs were included in the design of AYA-targeted programs, several cancer-specific studies have demonstrated that patient/AYA involvement in program design, co-creation, and stakeholder engagement leads to improved uptake, usability, relevance, and implementation of such programs [38,39,40]. Future studies and sites developing programming may wish to consider the importance of including patient partners in the development of programming and services, as well as leverage existing knowledge of successful AYA program implementation [41].

While this study has numerous strengths, its limitations must also be acknowledged. Overall, the survey response rate for the study from major pediatric and adult cancer centres was high. However, responses from Quebec were low, similar to the previous environmental scan conducted in 2011 [14]. Ramphal and colleagues hypothesized that this may have been attributed to their survey not being available in French. To reduce this limitation in this study, researchers translated the survey into French with little improvement in response rate. Continuing difficulties with response rates may be related to a significant strain on the healthcare system in Quebec, which is provincially regulated and has long struggled with a shortage of medical personnel and primary health providers [42]. This may have contributed to limited capacity to respond to research initiatives. Finally, in this study, key informants self-identified whether services were AYA-specific, which may have led to discrepancies and differing interpretations of what qualifies as an AYA service among the different sites.

Further, although attempts were made to identify the most appropriate informant at each institution, it is possible that there may have been someone in a better position to complete the survey, as a few participants indicated being unsure or not knowing about certain aspects of programming, funding, clinical trials, or services available at their institution. This may also have been attributable to the diversity of questions compromising the survey, some of which research staff may have been best positioned to answer such as questions regarding clinical trials, management regarding funding, and healthcare practitioners for available programs. Of note, this may also speak to some lack of awareness within centres around AYA-specific resources, such as available clinical trials, and may point to a need for centres to improve staff communication and education. Lastly, regional cancer centres were not contacted for this environmental scan and some are offering or beginning to implement some AYA programs or services [43].

## 5. Conclusions and Future Directions

In conclusion, this environmental scan identified that AYA services have improved across Canada over the last several years, including increased funding, organizational support, and available services/programming. However, these services continue to be limited and inconsistent, with significant inequities of services and programs across Canada, especially in Atlantic Canada. To address these gaps and promote equity across the country, a national AYA cancer strategy is essential, establishing common standards and guiding principles, while allowing provinces flexibility to tailor implementation to their unique circumstances. Key elements of this strategy could include: (1) collecting metrics on service availability and outcomes to benchmark progress and strengthen advocacy for sustainable funding beyond philanthropic sources; (2) implementing routine, standardized AYA-specific training for healthcare professionals to build capacity; and (3) fostering collaboration and resource sharing between pediatric and adult centres through formal transition pathways supported by hospital administrators. Additional priorities involve expanding access to psychosocial, survivorship, palliative care, fertility, and sexual health services and reducing barriers to clinical trial enrollment. Together, these steps offer a practical roadmap for delivering equitable, developmentally tailored care nationwide.

## Figures and Tables

**Table 1:** Pediatric and adult cancer centres contacted for the current study.

Province/Territory	Pediatric Centres (*n* = 16)	Adult Centres (*n* = 23)
British Columbia	BC Children’s Hospital	BC Cancer Agency
Alberta	Alberta Children’s Hospital Stollery Children’s Hospital	Arthur Child Comprehensive Cancer Centre (formerly Tom Baker Cancer Centre) The Cross Cancer Institute
Saskatchewan	Jim Pattison Children’s Hospital	Saskatoon Cancer Centre
Manitoba	CancerCare Manitoba	CancerCare Manitoba
Ontario	The Hospital for Sick Children Children’s Hospital of Eastern Ontario McMaster Children’s Hospital Children’s Hospital, London Health Sciences Centre Kingston Health Sciences Centre	Princess Margaret Cancer Centre Mount Sinai Hospital Sunnybrook Health Sciences Centre The Ottawa Hospital Juravinski Cancer Centre Kingston Health Sciences Centre London Health Sciences Centre Health Sciences North
Quebec	CHU de Sherbrooke CHU Sainte-Justine CHU de Quebec-Université Laval Montreal Children’s Hospital	Le Centre intégré de cancérologie du CHUM Jewish General Hospital Le Centre intégré de cancérologie de Laval CHUQ—Hôtel-Dieu de Quebec Cedars Cancer Centre
Newfoundland	Janeway Children’s Health & Rehabilitation Centre	Dr. H. Bliss Murphy Cancer Centre
New Brunswick	None	Dr. Everett Chalmers Regional Hospital
Nova Scotia	IWK Health Centre	QEII Cancer Centre
PEI	None	PEI Cancer Treatment Centre
Yukon	None	Whitehorse General Hospital

**Table 2:** Demographics of key informants who completed the environmental scan survey.

Hospital		Key Informant	N (%)
	Adult (*N* = 19)	Nurse	6 (32)
		Manager	5 (26)
		Oncologist	2 (11)
		Nurse Practitioner	1 (5)
		Palliative Care Physician	1 (5)
		Physician Assistant	1 (5)
		Psychologist	1 (5)
		Researcher	1 (5)
		Social Worker	1 (5)
	Pediatric (*N* = 13)	Oncologist	4 (31)
		Psychologist	3 (23)
		Manager	2 (15)
		Nurse	2 (15)
		Nurse Practitioner	1 (8)
		Social Worker	1 (8)

**Table 3:** Comparison of Ramphal et al. 2011 [14] and current study findings on AYA-specific services and programs in Canada.

	Ramphal et al. 2011 [14]		Current Study	
AYA Resource, Service, or Programme	Pediatric (*n* = 16)	Adult (*n* = 25)	Pediatric (*n* = 13)	Adult (*n* = 19)
AYA-specific programs for school-related/work issues	44%	17%	46%	32%
AYA-specific service for fertility concerns	25%	17%	54%	47%
AYA resources for sexual health	13%	8%	31%	37%
AYA-specific palliative care resources	13%	17%	46%	21%
AYA inpatient space	25%	13%	31%	5%
Cross-appointed staff	30%	20%	23%	32%

## Data Availability

The raw data supporting the conclusions of this article will be made available by the authors on reasonable request.

## Supplementary Materials

The following supporting information can be downloaded at: https://www.mdpi.com/article/10.3390/curroncol33020068/s1, AYA Environmental Scan Survey.

## Abbreviations

The following abbreviation is used in this manuscript:

AYA	Adolescent and Young Adults
