# Supplementary material for: An Environmental Scan of Services for Adolescents and Young Adults Diagnosed with Cancer Across Canadian Pediatric and Adult Tertiary Care Centres
Source: Curr Oncol. 2026 Jan 24;33(2):68. doi: 10.3390/curroncol33020068 (PMC12940081; doi:10.3390/curroncol33020068)
Supplement: Supplementary file 1 [file curroncol-33-00068-s001.zip › curroncol-4062365-supplementary.pdf]

Supplementary Material

An environmental scan of services for adolescents and young adults diagnosed with cancer across Canadian pediatric and adult tertiary care centres

Table of Contents

AYA Environmental Scan Survey..2

AYA Environmental Scan Survey

Q1.1 Environmental Scan of Adolescent and Young Adult Cancer Programs and Services across Canada

Members of the Division of Psychosocial Oncology at the University of Calgary and Department of Psychosocial Oncology at the Tom Baker Cancer Centre are undertaking an environmental scan of programs and resources offered for adolescent and young adult (AYA) cancer patients at pediatric and adult tertiary care hospitals across Canada.

You are being invited to complete this survey on behalf of your institution.
Each year, over 9000 adolescent and young adults are diagnosed with cancer in Canada and require specialized care and services. AYAs have been defined as cancer patients between the ages of 15-39, however institutions across Canada have different patient age requirements for AYA services. Addressing the unmet needs of the AYA population has been deemed a priority in Canada.

This environmental scan aims to: Create a database of all AYA programming/resources offered across Canada. Identify areas of strength and weakness of AYA programming across Canada. Identify gaps and barriers to AYA programming to inform implementation efforts. Promote implementation efforts and high-quality research related to AYA programming and resources to improve patient care. Please answer questions accurately and provide as much detail as possible for us to get a comprehensive picture of AYA programming/resources available across Canada. This questionnaire has several sections covering the following topics: General AYA programming including Personnel, Services, Environment, and Funding, and specific areas of interest such as Fertility/Sexual Health, Palliative Care, Return to work/school and Cancer-related Fatigue. Where necessary, a member of the project team may contact you by telephone to supplement or clarify data obtained on the form. We plan to compile tables of the findings. Detailed tables will be offered to leaders at each site. Results from this survey may be published in a peer-reviewed journal. If you have any questions or concerns you may contact the project team: Nicole Rutkowski at nicole.rutkowski@albertahealthservices.ca

You are consenting to participate in this quality improvement initiative by proceeding with the survey.

Q2.1 Please provide us with the following information:

- Institution Name ____
- Name of person completing the survey_________________________________
- Position/Title ____
- Telephone ____
- Email ____

Q2.2 Are there any other team members that we may benefit from contacting for additional information, if required? (e.g., a clinical lead, supervisor, etc.)

- Name ____
- Institution ____
- Position/Title ____
- Telephone ____
- Email ____

Q2.3 Are there any other team members that we may benefit from contacting for additional information, if required? (e.g., a clinical lead, supervisor, etc.)

- Name ____
- Institution ____
- Position/Title ____
- Telephone ____
- Email ____

Q2.4 Are there any other team members that we may benefit from contacting for additional information, if required? (e.g., a clinical lead, supervisor, etc.)

- Name ____
- Institution ____
- Position/Title ____
- Telephone ____
- Email ____

Q3.1 AYA Programming and Services

Q3.2 Does your institution offer AYA-specific resources and services?

- Yes
- No

Q3.3 Are AYA-specific resources and programs being developed?

- Yes
- No

Q3.4 Please describe the types of AYA-specific resources and programs your institution is working on developing:

____

Q3.5 Please explain why your institution is not developing or providing AYA-specific resources and programs:

____

Q4.1 Has AYA programming and service development been deemed a priority at your institution?

- Yes
- No

Q4.2 Please describe how AYA programming and service development has been prioritized at your institution:

____

Q5.1 Which cities or regions does your institution serve?

____

Q5.2 What is the lowest age your institution considers for AYA programming?

____

Q5.3 What is your institution's upper age limit for patients accessing AYA services?

____

Q5.4 How do you communicate about programming and resources with your AYA patients? Select all that apply.

- Phone
- Email
- Text
- Social Media (e.g., Instagram, Facebook)
- Newsletter
- Website
- Other, please describe ____

Q6.1 **Training and Collaboration**

Q6.2 Do you offer any services in collaboration with a pediatric or adult hospital for AYA patients?

- Yes
- No

Q6.3 Please describe the services you offer in collaboration:

____

Q6.4 Do you have staff who work across pediatric and adult hospitals for AYA patients?

- Yes
- No

Q6.5 Please describe the staff that sees AYA patients across adult and pediatric hospitals:

____

Q6.6 Please indicate which barriers have been encountered related to having AYA staff care for patients across adult/pediatric centres? Select all that apply.

- Funding
- Training opportunities to specialize in AYA care
- Lack of organizational support
- Logistics of having staff work between different institutions
- Environmental (e.g., rural setting)
- Other, please describe: ____

Q6.7 Does your institution offer specialized training on caring for AYA patients to staff?

- Yes
- No

Q6.8 Please describe the types of specialized AYA training you offer:

____

Q7.1 **Language of Services**

Q7.2 Are your AYA services offered in English, French, or both languages?

- English
- French
- Both languages equally
- Mostly English, with some services offered in French
- Mostly French, with some services offered in English

Q8.1 **Distress Screening**

Q8.2 Does your institution routinely screen for distress among AYA patients?

- Yes
- No

Q8.3 Please describe how your institution routinely screens for distress among AYA patients:

____

Q9.1 **Caregiver Support for AYA patients**

Q9.2 Does your institution offer family and caregiver support for AYA patients?

- Yes
- No

Q9.3 Please describe the types of supports you provide for family and caregivers of AYAs:

____

Q10.1 **Access to Clinical Trials**

Q10.2 Access to clinical trials has been identified as a barrier for AYAs in Canada. What is your institution's experience with connecting AYAs with relevant clinical trials?

____

Q10.3 Please indicate any barriers your institution has encountered related to connecting AYAs with relevant trials. Select all that apply.

- Clinical trials not available on site
- Lack of knowledge around available trials
- Lack of time
- Difficulty identifying trials for rare cancers
- Other, please describe ____

Q11.1 **Personnel**

Q11.2 Does your institution employ AYA-specific staff?

- Yes
- No

Q11.3 Which of the following types of personnel does your program employ for AYA services? Select all that apply.

- Clinical Psychologist
- Neuropsychologist
- Social worker
- School/work transitions Counsellor
- AYA patient navigator
- Clinical Nurse Specialist
- Program Coordinator
- Medical Director
- Physician
- Psychiatrist
- Research Staff
- Other, please describe: ____

Q12.1 **Services**

Q12.2 Which of the following services do you offer specifically for AYAs? Select all that apply.

- Educational class/webinars
- Newly diagnosed class
- AYA patient navigator
- AYA support group
- Individual counselling
- Community resources packages
- Newsletter
- Social media account
- Other, please describe: ____

Q12.3 If your institution provides educational classes/webinars and/or has an AYA navigator, please briefly describe the nature of these services:

____

Q13.1 **AYA Designated Spaces**

Q13.2 Does your institution offer a designated space for inpatient AYAs who are undergoing treatment? (e.g., lounge, outdoor space)

- Yes
- No

Q13.3 Please describe what type of designated space is available for inpatient AYAs:

____

Q13.4 Does your institution offer a designated space for outpatient AYAs who have finished treatment for programming?

- Yes
- No

Q13.5 Please describe what type of designated space is available for outpatient AYA programming:

____

Q14.1 **Funding for AYA Services**

Q14.2 How are your AYA programs funded? Select all that apply.

- Healthcare funds for the provision of AYA services
- Philanthropic donations
- University funds
- Research Grants/Government Grants
- Primarily healthcare funds, supplemented by donations
- Primarily donations, supplemented by healthcare funds
- No funding
- I don't know
- Other, please describe: ____

Q15.1 **Fertility**

Q15.2 Does your institution have specific services for addressing fertility concerns/preservation among AYA patients?

- Yes
- No

Q15.3 Which of the following does your institution provide regarding fertility concerns? Select all that apply.

- Resources on onco-fertility
- Support groups
- Referral to fertility clinics
- Educational class on onco-fertility
- Individual counselling with social work
- Individual counselling with Psychology
- Other, please describe: ____

Q15.4 Are fertility preservation options discussed with all AYA patients?

- Yes
- No

Q15.5 Who most often discusses fertility preservation with AYA patients? (e.g., nurse, oncologist, social work)

____

Q15.6 Do you discuss fertility preservation options with AYAs under the age of 18?

- Yes
- No

Q15.7 Please describe any barriers your institution has encountered related to discussing fertility with AYAs:

____

Q15.8 Are there any gaps in services at your institution regarding fertility concerns and preservation for AYAs?

____

Q16.1 **Sexual Health**

Q16.2 Does your institution have specific services for addressing sexual health concerns among AYA patients?

- Yes
- No

Q16.3 Which of the following does your institution provide regarding sexual health concerns specifically for AYAs? Select all that apply.

- Resources on sexual health
- Support groups
- Educational class on sexual health
- Individual counselling with social work
- Individual counselling with Psychology
- Individual counselling with sexual health consultant
- Other, please describe: ____

Q17.1 **Palliative Care**

Q17.2 Does your institution have specific services for addressing palliative care and end-of-life concerns among AYA patients?

- Yes
- No

Q17.3 Which of the following does your institution provide for palliative care and end-of-life concerns specifically for AYA patients? Select all that apply.

- Resources on end of life
- Support groups
- Educational class
- Individual counselling with social work
- Individual counselling with Psychology
- Spiritual counselling
- Other, please describe: ____

Q17.4 If you selected AYA specific resources and/or educational class on end of life, could you please briefly describe these resources/programs?

____

Q18.1 **Return to Work/School**

Q18.2 Does your institution have specific services for addressing return to work/school for AYA patients?

- Yes
- No

Q18.3 Please describe the type of services you provide to address return to work/school:

____

Q19.1 **Cancer-Related Fatigue**

Q19.2 Does your institution have specific services for addressing cancer-related fatigue in AYA patients?

- Yes
- No

Q19.4 Which of the following does your institution provide regarding cancer-related fatigue for AYAs? Select all that apply.

- Resources on cancer-related fatigue
- Support groups
- Educational class
- Individual counselling with social work
- Individual counselling with Psychology
- Vocational/return to work counselling
- Other, please describe: ____

Q19.5 If you selected AYA specific resources and/or educational class on cancer-related fatigue, could you please briefly describe these resources/programs?

____

Q19.3 Does your institution routinely screen for cancer-related fatigue among AYA patients?

- Yes
- No

Q78 **General Feedback**

Q20.1 What are the top strengths of your AYA programs and services?

____

Q20.2 What are some areas your institution would like to improve regarding AYA programs and services?

____

Q20.3 Any other comments or information you believed would be helpful for us to capture regarding your institution's AYA programming or services which we have not asked about?

____

Q20.4 We thank you for the time and effort you have put into completing this survey. Your contributions will help improve patient care for AYAs across Canada. Please click to the next page to submit your survey.

End of Survey
